# Constructive, Not Destructive, Therapeutics

**Published:** 1910-04

**Authors:** 


### Abstracts and Selections.

### Constructive, not Destructive, Therapeutics.

There is a conviction among physicians of today that the prolonged administration of such drugs as the bromides, hyoscyamus and valerian in cases of nervouse disturbances attending the termination of the menstrual function is highly detrimental to the future welfare of the patient. The opinion is now general that such treatment is destructive rather than constructive in nature.

It is now held that, inasmuch as the mental states, such as hysteria and melancholia, which are frequently manifested at this period of life, have for their exciting cause alterations of the sexual system, it is proper that their relief be accomplished by the employment of agents that exert an influence directly on these parts.

It is conceded that the sedatives mentioned do afford a certain measure of palliation, but experience seems to have proved that they are ultimately injurious rather than beneficial in action.

The use of utero-ovarian stimulants, particularly those which are primarily antispasmodic in action, has been so uniformly more satisfactory than general sedatives that the employment of the former is now urged by our most eminent practitioners.

If, at the approach of the menopause, such an agent as Ergoapiol (Smith) is administered with due regularity, irritability of the sexual system and disturbance of the nervous system will be prevented. Under such treatment, normal atrophy of the reproductive organs takes place without the development of the neurotic disturbances commonly associated with this physiologic change of life.

On account of its antispasmodic and tonic action on the female reproductive organs, Ergoapiol (Smith) is particularly serviceable in instances where the menopause is being approached by women of nervous temperaments.

Under the influence of this preparation the so-called "change of life" is relieved of its characteristic discomforts. The mental as well as the physical state of the individual is benefited by the administration of Ergoapiol (Smith) in doses of one capsule three times each day.

